# Fruit and vegetable consumption before and during pregnancy and birth weight of new-borns in Japan: the Tohoku medical megabank project birth and three-generation cohort study

**DOI:** 10.1186/s12937-020-00595-z

**Published:** 2020-08-03

**Authors:** Yudai Yonezawa, Taku Obara, Takahiro Yamashita, Junichi Sugawara, Mami Ishikuro, Keiko Murakami, Aoi Noda, Fumihiko Ueno, Shigenori Suzuki, Hiroyuki Suganuma, Shinichi Kuriyama

**Affiliations:** 1 Tohoku Medical Megabank Organization, Tohoku University, Sendai, Japan; 2 Innovation Division, KAGOME CO., LTD., Nasushiobara, Japan; 3 Division of Molecular Epidemiology, Tohoku University Graduate School of Medicine, Sendai, Japan; 4 Department of Pharmaceutical Sciences, Tohoku University Hospital, Sendai, Japan; 5 Graduate School of Medicine, Tohoku University, Sendai, Japan; 6 International Research Institute of Disaster Science, Tohoku University, Sendai, Japan

**Keywords:** Fruit, Vegetables, Pregnancy, Birth weight

## Abstract

**Background:**

Associations of fruit and vegetable consumption before and during pregnancy with birth weight of new-borns and the risk of low birth weight (LBW) remain unclear.

**Methods:**

Between July 2013 and March 2017, we recruited 23,406 pregnant women in the Tohoku Medical Megabank Project Birth and Three-Generation Cohort Study (TMM BirThree Cohort Study). Fruit and vegetable consumption before and during pregnancy was calculated using food frequency questionnaires. Information regarding birth weight was obtained from medical records, and LBW was defined as < 2500 g. We used a multivariable linear regression model and a multivariate logistic regression model to assess associations between fruit and vegetable consumption and birth weight/risk of LBW.

**Results:**

In total, 17,610 women were included in the analysis. Mean birth weight was 3061.8 ± 354.1 g, and 5.4% of the new-borns had LBW. Compared to women in the lowest quartile of fruit consumption between pre- and early pregnancy, women in the highest quartile had heavier new-borns (β = 49.4; 95% CI: 34.1–64.7) and lower risk of LBW (OR: 0.79; 95% CI: 0.65–0.95). Women in the highest quartile of fruit consumption from early to mid-pregnancy also had heavier new-borns (β = 32.3; 95% CI: 17.1–47.6), and they tended to have lower risk of LBW (OR: 0.83; 95% CI: 0.69–1.01). Results of analysing the association between changes in fruit consumption from pre- to mid-pregnancy and birth outcomes revealed that women with continuous high fruit consumption from pre- to mid-pregnancy had heavier new-borns (β = 37.6; 95% CI: 25.0–50.3), but they did not have lower risk of LBW (OR: 0.90; 95% CI: 0.77–1.06). Associations involving vegetable consumption and birth weight/risk of LBW were not observed.

**Conclusions:**

Fruit consumption before and during pregnancy was positively associated with birth weight of new-borns and negatively associated with risk of LBW.

## Background

Low birth weight (LBW, < 2500 g) contributes to approximately 80% of neonatal deaths worldwide [1, 2]. Surviving LBW new-borns have a high risk of obesity, hypertension, insulin resistance, and type 2 diabetes later in life [3–5]. From 1980 to 2015, the prevalence of LBW increased from 5.2 to 9.5% in Japan [6], and that prevalence was 1.9% higher than the average prevalence of 7.6%, in other high-income countries [7]. This indicates that LBW is a serious problem in Japan.

Dietary habits before and during pregnancy are one of the major factors related to birth weight of new-borns [8, 9]. In particular, single or mixed supplementation of iron, folic acid, and magnesium is reported to be positively associated with birth weight [10–12], and is associated with decreased risk of LBW [13, 14]. Also, positive associations involving vitamin C consumption during pregnancy and birth weight have been reported [15]. In the US, Canada, and many other countries, several foods are fortified with folate and iron [16]. However, this is not the case in Japan, and Japanese pregnant women need to compensate for deficiency of those micronutrients. We hypothesized that fruits and vegetables, rich sources of such micronutrients, have a positive effect on birth weight.

To our knowledge, 10 previous studies have investigated associations between fruit and vegetable consumption, before or during pregnancy, and birth weight. Only one study researched fruit and vegetable consumption before pregnancy [17]. The results of this cohort study in the US were limited because of the small number of participants included in the analysis (*n* = 115), and using simple questions that asked whether fruits/vegetables were consumed at least once a day or not. Among the remaining nine studies, six suggested that fruit and/or vegetable consumption during pregnancy is positively associated with birth weight of new-borns [12, 18–22]. In contrast, three studies reported that fruit and vegetable consumption during pregnancy is not significantly associated with birth weight [23–25]. Additionally, several previous studies reported that women change their fruit and vegetable consumption from pre-pregnancy to pregnancy [26, 27]. However, no studies have evaluated fruit and vegetable consumption before and during pregnancy at the same time, and this may be one of the reasons for inconsistent results among previous reports. We hypothesised that changes in fruit and vegetable consumption from before to during pregnancy affect new-born birth weight.

This study aimed to clarify associations involving fruit and vegetable consumption before and during pregnancy, and birth weight of new-borns/risk of LBW. Moreover, we analysed associations between changes in fruit and vegetable consumption before and during pregnancy and birth weight of new-borns/risk of LBW to clarify the appropriate period of fruit and vegetable consumption before and during pregnancy.

## Methods

### Study design and population

**Fig. 1:**
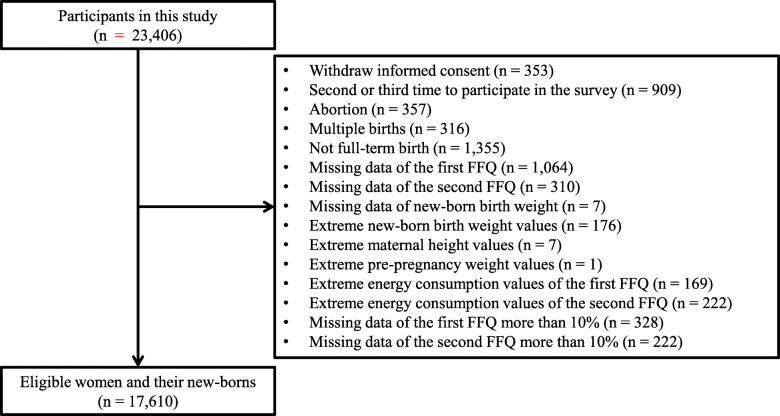
Flow chart of participant exclusion criteria in this study. The flow chart shows the exclusion criteria and the number of total participants, excluded participants, and eligible participants

This study was based on data obtained by the Tohoku Medical Megabank Project Birth and Three-Generation Cohort Study (TMM BirThree Cohort Study). The TMM BirThree Cohort Study is a prospective cohort study based in Miyagi Prefecture, Japan. Detailed information regarding the TMM BirThree Cohort Study has been reported elsewhere [28, 29]. In total 23,406 pregnant women, including multiple different pregnancies, were recruited between July 2013 and March 2017. Of these participants, women who withdrew informed consent (*n* = 353), women who participated in the survey for the second or third time (*n* = 909), women who suffered abortions (*n* = 357), and women who experienced multiple births (*n* = 316), were excluded. New-borns who were not full-term at birth (< 37 weeks or ≥ 42 weeks, *n* = 1355), women for whom there were no data corresponding to the first food frequency questionnaire (FFQ) (*n* = 1064) and the second FFQ (*n* = 310) were also excluded. Among the remaining participants, new-borns whose birth weight data were missing (*n* = 7), new-borns who exhibited extreme birth weight values (< 1000 g or ≥ 4000 g, *n* = 176), women who had extreme height values (< 100 cm, *n* = 7), women who had extreme pre-pregnancy weight values (< 20 kg, *n* = 1), were excluded. We also excluded women who had extreme energy consumption values identified in the first and second FFQs (< 500 kcal or ≥ 6500 kcal, *n* = 169 and 222, respectively), and women who had missing data (10%) corresponding to the first or second FFQ (*n* = 328 and 222). Finally, 17,610 mother-newborn pairs were included in the analysis (Fig. 1).

### Exposure variables

The first and second FFQs were used to evaluate dietary consumption during two different time periods. We defined early pregnancy as 0 to 13 weeks of gestation, and mid-pregnancy as 14 to 27 weeks of gestation. The first FFQ was given in early pregnancy and queried the frequency and amount of food items and beverages over the past year to evaluate dietary consumption from pre- to early pregnancy. The second FFQ was given in mid-pregnancy and identified the frequency and quantity of food items and beverages consumed since the first FFQ was answered so as to evaluate dietary consumption from early to mid-pregnancy. Average response periods of the first and second FFQs were 20.2 ± 7.4 weeks and 28.3 ± 5.4 weeks of gestation, respectively. Frequency and amount of 130 food items and beverages consumption were evaluated through each FFQ. The first and second FFQs involved 10 response options: “constitutionally unable to eat it”, “never or less than once a month”, “from one to three times a month”, “once or twice a week”, “three or four times a week”, “five or six times a week”, “once a day”, “two or three times a day”, “from four to six times a day”, and “seven or more times a day”. Portion sizes were defined, and the amounts were answered with response options as follows: “less than half”, “the same”, and “more than one and a half times”. Frequency and amount of food items and beverages were converted into average daily consumption by multiplying frequency and quantity. Those items included 18 fruit portions (papaya; mandarin; other citrus; apple; persimmon; strawberry; grape; melon; watermelon; peach; pear; kiwi; pineapple; banana; fruit jam; orange juice; apple juice; pickled plum) and 26 vegetable items (carrot; spinach; pumpkin; cabbage; radish; pickled radish; pickled green leaf; pickled Chinese cabbage; pickled cucumber; pickled eggplant; green pepper; tomato; green onion; Chinese chives; shungiku; Japanese mustard spinach; broccoli; onion; cucumber; eggplant; Chinese cabbage; burdock; bean sprout; green bean; lettuce; tomato juice). Total fruit and vegetable consumption were identified by summing responses to fruit and vegetable consumption queries. Fruit and vegetable consumption were included in the analysis as continuous or categorical values which were divided into quartiles. Total energy and nutrient consumption were calculated using the Standard Tables of Food Composition in Japan (Fifth Revised and Enlarged Edition 2005) [30]. Each food and nutrient consumed was energy-adjusted using the residual method [31]. Also, we defined four categories based on changes in total fruit and vegetable consumption from pre- to mid-pregnancy. The definitions of the four categories were described as follows: women whose answers to the first and second FFQs were in the first or second quartile (Category A), women whose answers to the first FFQ comprised the first or second quartile and whose responses to the second FFQ were in the third or fourth quartile (Category B), women whose answers to the first FFQ were in the third or fourth quartile and whose responses to the second FFQ were the first or second quartile (Category C), women whose answers to the first and second FFQs were in both the third or fourth quartile (Category D).

### Outcome variables

Birth weight of new-borns and LBW were used as outcome variables. Information concerning birth weight (g) was obtained from birth records. LBW was defined as birth weight < 2500 g [32].

### Confounders

Confounders considered to affect exposures and outcomes were selected from previous reports [9, 12, 18–22]. Information regarding maternal age (years) and parity (never, one, or more) were gathered from medical records, and height (cm) and pre-pregnancy weight (kg) were self-reported values. Pre-pregnancy body mass index (BMI) (kg/m^2^) was calculated by dividing pre-pregnancy weight (kg) by the square of height (m^2^). BMI values were divided into three categories (< 18.5, 18.5 ≤ BMI < 25.0, ≥ 25.0 kg/m^2^). Information on cigarette smoking (never, stopped before pregnancy, stopped after pregnancy, current), alcohol consumption (never, ever, current), folic acid supplementation during early pregnancy (yes/no) was gathered from the questionnaire during early pregnancy. Information concerning household income (< 4,000,000, 4,000,000-5,999,999, ≥ 6,000,000 Japanese Yen/year) was gathered from the questionnaire during mid-pregnancy. Maternal educational attainment data (high school graduate or less, junior college or vocational college graduate, university graduate or above, others) were gathered a year after birth. Total meat, fish, grain, potato, beans, and dairy product consumption were identified by the same method as for fruit and vegetable consumption and were divided into quartiles. Changes in dietary consumption of these food products were also characterised, and confounders were imputed by multivariate imputation methods by chained equations (MICE) assuming missing data as random [33]. For the present study, MICE imputed each incomplete confounder by generating plausible synthetic values given exposure, outcome, and the other confounders in the data. We independently analysed 20 copies of the data, each with missing values imputed in the multivariate analyses.

### Statistical analysis

Continuous variables were expressed as means ± standard deviation (SD), and categorical variables were indicated as frequencies and percentages. Association between fruit and vegetable consumption and birth weight of new-borns were evaluated by multivariable linear regression analysis to calculate regression coefficients and 95% confidence intervals (95% CI) per quartile. Also, associations involving fruit and vegetable consumption and risk of LBW were evaluated by multivariate logistic regression analysis to calculate odds ratio (OR) and 95% CI per quartiles. Associations between changes in fruit and vegetable consumption from pre-pregnancy to mid-pregnancy and birth weight of new-borns/risk of LBW were also analysed by multivariable linear regression analysis/multivariate logistic regression analysis. We used the crude model and the multivariable model adjusted confounders. First quartile and category A were used as a reference group. *P* <  0.05 was considered statistically significant. Statistical analyses were performed using R, v.3.6.0.

## Results

### Characteristics of study population

Study participants had a mean age of 31.8 ± 4.9 years old, mean height of 158.4 ± 5.3 cm, and mean pre-pregnancy weight of 54.2 ± 9.2 kg (Table 1). Pre-pregnancy BMI measurements revealed that 13.2% of women were underweight (BMI < 18.5 kg/m^2^), 72.8% were normal weight (18.5 ≤ BMI < 25.0 kg/m^2^), 12.6% of women were overweight (BMI ≥ 25.0 kg/m^2^), and 1.4% of data was missing. Approximately half of the participants were experiencing their first birth. The number of missing data points regarding maternal educational attainment was large (*n* = 6120) because that information was gathered a year after birth. Totally 16.1% of the participants smoked when they became pregnant. A large proportion of women (19.8%) drank alcohol and around half (56.2%) took folic acid supplements during pregnancy. Dietary consumption from early to mid-pregnancy was lower than from pre- to early pregnancy except for dairy product consumption. Mean birth weight was 3061.8 ± 354.1 g and 5.4% of the new-borns were categorised as LBW. Focusing on protein, fat, and carbohydrate consumption, women with high fruit consumption had high carbohydrate consumption, and women with high vegetable consumption had high protein and fat consumption (Table S1-S4). Iron, folate, magnesium, and vitamin C consumption became higher per quartile of both fruit and vegetable consumption increase (Table S1-S4).

**Table 1:** Characteristics of pregnant women and new-borns

	Mean ± SD or n (%)
n	17,610
General characteristics	
Maternal age (years)	31.8 ± 4.9
Height (cm)	158.4 ± 5.3
Pre-pregnancy weight (kg)	54.2 ± 9.2
Pre-pregnancy BMI^a^	
< 18.5 (kg/m^2^)	2325 (13.2)
18.5 ≤ BMI < 25.0 (kg/m^2^)	12,828 (72.8)
≥ 25.0 (kg/m^2^)	2213 (12.6)
Missing	244 (1.4)
Parity	
Never	8011 (45.5)
One or more	9570 (54.3)
Missing	29 (0.2)
Educational attainment	
High school graduate or less	3685 (20.9)
Junior college or vocational college graduate	4392 (24.9)
University graduate or above	3311 (18.8)
Others	26 (0.1)
Missing	6196 (35.2)
Household income	
< 4,000,000 (Japanese Yen/year)	6069 (34.5)
4,000,000-5,999,999 (Japanese Yen/year)	5481 (31.1)
≥ 6,000,000 (Japanese Yen/year)	5263 (29.9)
Missing	797 (4.5)
Cigarette smoking	
Never	10,639 (60.4)
Stoped before pregnancy	4078 (23.2)
Stoped after pregnancy	2437 (13.8)
Current	400 (2.3)
Missing	56 (0.3)
Alcohol consumption	
Never	8011 (45.5)
Former	6078 (34.5)
Current	3480 (19.8)
Missing	41 (0.2)
Folic acid supplementation during pregnancy	
Yes	9904 (56.2)
No	7678 (43.6)
Missing	28 (0.2)
Dietary and nutrient consumption (From pre- to early pregnancy)^b^	
Total fruit consumption (g/d)	152.1 ± 142.2
Total vegetable consumption (g/d)	155.3 ± 112.9
Total meat consumption (g/d)	77.1 ± 46.8
Total fish consumption (g/d)	37.4 ± 34.3
Total grain consumption (g/d)	426.7 ± 120.2
Total potato consumption (g/d)	24.5 ± 19.1
Total bean consumption (g/d)	59.6 ± 62.8
Total dairy product consumption (g/d)	202.7 ± 241.2
Maternal total energy consumption (kcal)	1686.2 ± 591.1
Protein consumption (g/d)	57.6 ± 11.1
Fat consumption (g/d)	57.7 ± 14.3
Carbohydrate consumption (g/d)	213.2 ± 37.4
Iron consumption (mg/d)	6.5 ± 1.7
Folate consumption (μg/d)	258.8 ± 99.2
Magnesium consumption (mg/d)	215.4 ± 46.4
Vitamin C consumption (mg/d)	83.5 ± 46.2
Dietary and nutrient consumption (From early to mid pregnancy)^b^	
Total fruit consumption (g/d)	146.8 ± 129.0
Total vegetable consumption (g/d)	146.5 ± 92.5
Total meat consumption (g/d)	74.4 ± 41.7
Total fish consumption (g/d)	35.6 ± 28.1
Total grain consumption (g/d)	408.2 ± 110.2
Total potato consumption (g/d)	23.9 ± 18.3
Total bean consumption (g/d)	56.0 ± 60.4
Total dairy product consumption (g/d)	224.4 ± 250.2
Maternal total energy consumption (kcal)	1612.4 ± 560.9
Protein consumption (g/d)	56.0 ± 10.0
Fat consumption (g/d)	55.9 ± 12.8
Carbohydrate consumption (g/d)	204.1 ± 33.0
Iron consumption (mg/d)	6.2 ± 1.6
Folate consumption (μg/d)	244.0 ± 83.9
Magnesium consumption (mg/d)	205.8 ± 41.8
Vitamin C consumption (mg/d)	78.2 ± 40.5
Outcomes of infants	
Birth weight (g)	3061.8 ± 354.1
LBW	
< 2500 (g)	951 (5.4)

^a^BMI was calculated by dividing the pre-pregnancy weight (kg) by the square of height (m^2^)

^b^Energy-adjusted using the residual method except for maternal total energy consumption

*BMI* Body mass index; *LBW* Low birth weight; *SD* Standard deviation

### Energy-adjusted fruit and vegetable consumption from pre- to mid-pregnancy and new-born birth weight

In both crude and multivariable models, higher fruit consumption from pre- to early pregnancy was associated with the heavier birth weight of new-borns (Table 2). In addition, higher fruit consumption from early to mid-pregnancy was also associated with heavier new-born birth weight. The adjustment of confounders did not significantly affect the results of both fruit consumption from pre- to early pregnancy and that from early to mid-pregnancy and birth weight. Both vegetable consumption from pre- to early pregnancy and that from early to mid-pregnancy were not associated with birth weight of new-borns.

**Table 2:** Energy-adjusted fruit and vegetable consumption from pre- to mid-pregnancy and new-born birth weight

	From pre- to early pregnancy				From early to mid-pregnancy			
Quartiles	Crude model		Multivariable model^a^		Crude model		Multivariable model^a^	
	β	95% CI	β	95% CI	β	95% CI	β	95% CI
Fruit^b^								
Continues	15.5	10.9–20.2	16.9	12.0–21.7	9.9	5.2–14.6	10.3	5.5–15.1
Categorical								
First quartile	Reference		Reference		Reference		Reference	
Second quartile	12.3	−2.5–27.1	14.3	−0.8–29.4	4.4	−10.4–19.2	7.7	−7.3–22.7
Third quartile	33.6	18.8–48.4	36.5	21.3–51.8	13.8	−1.0–28.6	16.6	1.6–31.7
Fourth quartile	44.7	29.9–59.4	49.4	34.1–64.7	29.9	15.1–44.7	32.3	17.1–47.6
*P* for trend	< 0.001		< 0.001		< 0.001		< 0.001	
Vegetables^b^								
Continues	0.6	−4.1–5.2	−4.4	−9.6–0.7	2.1	−2.6–6.8	−0.1	−5.2–5.0
Categorical								
First quartile	Reference		Reference		Reference		Reference	
Second quartile	3.7	−11.1–18.5	−2.3	−17.7–13.0	−4.0	− 18.8–10.8	− 2.5	−17.7–12.7
Third quartile	2.0	−12.8–16.8	−8.7	−24.5–7.1	4.6	−10.2–19.4	4.8	−10.8–20.4
Fourth quartile	2.5	−12.3–17.2	− 12.5	−28.8–3.7	4.1	−10.7–18.9	−3.2	− 19.4–12.9
*P* for trend	0.81		0.09		0.38		0.97	

^a^Adjusted for maternal age (continuous variable), pre-pregnancy BMI (< 18.5; 18.5 to 25.0; ≥25.0 kg/m^2^), parity (never; one or more), educational attainment (high school graduate or less; junior college or vocational college graduate; university graduate or above; others), household income (< 4,000,000; 4,000,000-5,999,999; ≥ 6,000,000 Japanese Yen/year), cigarette smoking (never; stop before pregnancy; stop after pregnancy; current), alcohol drinking (never; former; current), folic acid supplementation during pregnancy (yes vs. no), total fruit, vegetable, meat, fish, grain, potato, bean, and daily product consumption (in quartiles) except for the exposures

All dietary consumption was energy-adjusted using the residual method

*P* for trends were calculated as trends across categories

*BMI* Body mass index; *95% CI* 95% confidence interval

### Energy-adjusted fruit and vegetable consumption from pre- to mid-pregnancy and LBW risk

Compared to the first quartile of fruit consumption from pre- to early pregnancy, other quartiles had a lower risk of LBW in both the crude (*P* = 0.004) and multivariable models (*P* = 0.01) (Table 3). In contrast, fruit consumption from early to mid-pregnancy was not associated with the risk of LBW, although ORs of the second and fourth quartiles were low. Both vegetable consumption from pre- to early pregnancy and that from early to mid-pregnancy were not associated with LBW risk.

**Table 3:** Energy-adjusted fruit and vegetable consumption from pre- to mid-pregnancy and LBW risk

	From pre- to early pregnancy				From early to mid-pregnancy			
Quartiles	Crude model		Multivariable model^a^		Crude model		Multivariable model^a^	
	OR	95% CI	OR	95% CI	OR	95% CI	OR	95% CI
Fruit								
First quartile	Reference		Reference		Reference		Reference	
Second quartile	0.81	0.68–0.97	0.81	0.68–0.98	0.88	0.73–1.06	0.88	0.72–1.06
Third quartile	0.75	0.62–0.90	0.76	0.62–0.92	1.03	0.86–1.23	1.01	0.84–1.22
Fourth quartile	0.78	0.65–0.93	0.79	0.65–0.95	0.84	0.69–1.01	0.83	0.69–1.01
*P* for trend	0.004		0.01		0.22		0.22	
Vegetables								
First quartile	Reference		Reference		Reference		Reference	
Second quartile	0.97	0.81–1.17	1.00	0.83–1.22	1.08	0.90–1.30	1.05	0.87–1.28
Third quartile	0.94	0.78–1.13	1.00	0.82–1.23	0.97	0.81–1.17	0.95	0.78–1.16
Fourth quartile	0.98	0.82–1.18	1.07	0.88–1.32	1.03	0.86–1.24	1.04	0.85–1.28
*P* for trend	0.78		0.57		0.97		0.97	

^a^Adjusted for maternal age (continuous variable), pre-pregnancy BMI (< 18.5; 18.5 to 25.0; ≥25.0 kg/m^2^), parity (never; one or more), educational attainment (high school graduate or less; junior college or vocational college graduate; university graduate or above; others), household income (< 4,000,000; 4,000,000-5,999,999; ≥ 6,000,000 Japanese Yen/year), cigarette smoking (never; stop before pregnancy; stop after pregnancy; current), alcohol drinking (never; former; current), folic acid supplementation during pregnancy (yes vs. no), total fruit, vegetable, meat, fish, grain, potato, bean, and daily product consumption (in quartiles) except for the exposures

All dietary consumption was energy-adjusted using the residual method

*P* for trends were calculated as trends across categories

*BMI* Body mass index; *LBW* Low birth weight; 95% CI 95% confidence interval; *OR* Odds ratio

### Changes in energy-adjusted fruit and vegetable consumption from pre- to mid-pregnancy and new-born birth weight

Women who had high fruit consumption only from pre- to early pregnancy (Category C) and women who had high fruit consumption from pre- to mid-pregnancy (Category D) more often had heavier babies, compared to women who had low fruit consumption from pre- to mid-pregnancy (Category A) (Table 4). Above all, women who had high fruit consumption from pre- to mid-pregnancy (Category D) had higher regression coefficients involving birth weight compared to women who had high fruit consumption only from pre- to early pregnancy (Category C). There was no difference in regression coefficients between women who had high fruit consumption only from early to mid-pregnancy (Category B) and women who had low fruit consumption from pre- to mid-pregnancy (Category A). Changes in vegetable consumption from pre- to mid-pregnancy were not associated with birth weight of new-borns.

**Table 4:** Changes in energy-adjusted fruit and vegetable consumption from pre- to mid-pregnancy and new-born birth weight

Quartiles		Crude model		Multivariable model^a^	
	n	β	95% CI	β	95% CI
Fruit					
Category A^b^	6266	Reference		Reference	
Category B^c^	2539	−2.6	−18.9–13.7	−5.1	−21.3–11.2
Category C^d^	2539	20.5	4.2–36.8	19.4	3.1–35.8
Category D^e^	6266	37.0	24.6–49.4	37.6	25.0–50.3
Vegetables					
Category A^b^	6348	Reference		Reference	
Category B^c^	2457	9.8	−6.7–26.3	6.3	−10.4–23.0
Category C^d^	2457	−0.9	−17.4–15.6	−9.6	−26.3–7.1
Category D^e^	6348	4.7	−7.7–17.0	−6.4	−19.8–6.9

^a^Adjusted for maternal age (continuous variable), pre-pregnancy BMI (< 18.5; 18.5 to 25.0; ≥25.0 kg/m^2^), parity (never; one or more), educational attainment (high school graduate or less; junior college or vocational college graduate; university graduate or above; others), household income (< 4,000,000; 4,000,000-5,999,999; ≥ 6,000,000 Japanese Yen/year), cigarette smoking (never; stop before pregnancy; stop after pregnancy; current), alcohol drinking (never; former; current), folic acid supplementation during pregnancy (yes vs. no), changes in fruit, vegetable, meat, fish, grain, potato, bean, and daily product consumption (category A; B; C; D) except for the exposures

^b^Women whose answers to the first and second FFQs were in the first or second quartile

^c^Women whose answers to the first FFQ comprised the first or second quartile and whose responses to the second FFQ were in the third or fourth quartile

^d^Women whose answers to the first FFQ were in the third or fourth quartile and whose responses to the second FFQ were the first or second quartile

^e^Women whose answers to the first and second FFQs were in both the third or fourth quartile

All dietary consumption was energy-adjusted using the residual method

*BMI* Body mass index; *FFQ* Food frequency questionnaire; *95% CI* 95% confidence interval

### Changes in fruit and vegetable consumption from pre- to mid-pregnancy and risk of LBW

Women who had high fruit consumption only from pre- to early pregnancy (Category C) had a low risk of LBW, compared to women who had low fruit consumption from pre- to mid-pregnancy in the crude model (Category A) (Table 5). In the multivariable model, women who had high fruit consumption only from pre- to early pregnancy (Category C) tended to have a low risk of LBW, though the difference was not statistically significant. Changes in vegetable consumption from pre-pregnancy to mid-pregnancy were not associated with the risk of LBW.

**Table 5:** Changes in energy-adjusted fruit and vegetable consumption from pre- to mid-pregnancy and LBW risk

Quartiles		Crude model		Multivariable model^a^	
	n	OR	95% CI	OR	95% CI
Fruit					
Category A^b^	6266	Reference		Reference	
Category B^c^	2539	1.03	0.85–1.25	1.03	0.85–1.26
Category C^d^	2539	0.78	0.63–0.96	0.81	0.65–1.00
Category D^e^	6266	0.88	0.76–1.03	0.90	0.77–1.06
Vegetables					
Category A^b^	6348	Reference		Reference	
Category B^c^	2457	0.95	0.77–1.17	0.95	0.77–1.17
Category C^d^	2457	0.98	0.79–1.20	1.03	0.83–1.27
Category D^e^	6348	0.96	0.82–1.12	0.99	0.83–1.17

^a^Adjusted for maternal age (continuous variable), pre-pregnancy BMI (< 18.5; 18.5 to 25.0; ≥25.0 kg/m^2^), parity (never; one or more), educational attainment (high school graduate or less; junior college or vocational college graduate; university graduate or above; others), household income (< 4,000,000; 4,000,000-5,999,999; ≥ 6,000,000 Japanese Yen/year), cigarette smoking (never; stop before pregnancy; stop after pregnancy; current), alcohol drinking (never; former; current), folic acid supplementation during pregnancy (yes vs. no), changes in fruit, vegetable, meat, fish, grain, potato, bean, and daily product consumption (category A; B; C; D) except for the exposures

^b^Women whose answers to the first and second FFQs were in the first or second quartile

^c^Women whose answers to the first FFQ comprised the first or second quartile and whose responses to the second FFQ were in the third or fourth quartile

^d^Women whose answers to the first FFQ were in the third or fourth quartile and whose responses to the second FFQ were the first or second quartile

^e^Women whose answers to the first and second FFQs were in both the third or fourth quartile

All dietary consumption was energy-adjusted using the residual method

*BMI* Body mass index; *FFQ *Food frequency questionnaire; *LBW* Low birth weight; 95% CI 95% confidence interval; *OR* Odds ratio

## Discussion

We found that fruit consumption from pre- to mid-pregnancy was positively associated with birth weight and negatively associated with the risk of LBW. Also, continuous high fruit consumption from pre- to mid-pregnancy resulted in increased birth weight of new-borns and partly decreased risk of LBW.

In this study, women with continuous high fruit consumption from pre- to mid-pregnancy (Category D) had new-borns with higher birth weights compared to women in other categories. This result indicated that continuous fruit consumption from pre-pregnancy positively affected birth weight. The importance of dietary consumption before pregnancy in terms of birth outcomes is gradually becoming clearer [34], whereas information regarding relationships involving dietary consumption before pregnancy and birth weight are extremely limited. A population-based cohort study in the US showed inconsistent results to those of our study in that not fruit, but rather vegetable consumption during preconception had a positive effect on birth weight [17]. As for LBW, women who had high fruit consumption only from pre- to early pregnancy (Category C) tended to have lower risk, though the difference was not statistically significant. A cross-sectional study in Australia reported that vegetarian dietary patterns before pregnancy bore no relation to the risk of LBW, and women with high protein/fruit patterns tended to have a low risk of LBW despite no statistically significant differences [35]. These results seem consistent with our study, but simple comparisons are not possible because of differences in the types of exposure [35]. Further studies would be needed to reveal the effects of continuous high fruit consumption from pre-pregnancy and the mechanisms involved.

This study revealed that women with high fruit consumption also had high carbohydrate consumption compared to women with high vegetable consumption (Table S1-S4). In a longitudinal study in Sri Lanka, carbohydrate consumption in the second trimester was positively associated with birth weight [36]. In our sub-analysis, carbohydrate consumption from pre- to early pregnancy and that from early to mid-pregnancy were associated with increasing birth weight of new-borns (Table S5). In addition, association between fruit consumption and new-born birth weight became slightly smaller by adjusting carbohydrate consumption (Table S6). These results may indicate that high carbohydrate consumption is one of the causes of the positive association between fruit consumption and birth weight, although this effect is small. Fruit contains various micronutrients such as iron, folic acid, magnesium, and vitamin C. Consumption of these four micronutrients increased in proportion to consumption of both fruit and vegetables (Table S1-S4). Among these micronutrients, only vitamin C consumption from pre- to early pregnancy was associated with birth weight of new-borns (Table S5). However, association between fruit consumption and birth weight of infants was hardly affected by adjusting to micronutrient consumption (Table S6). Therefore, these results may suggest that iron, folic acid, magnesium, and vitamin C consumption had little effect on association between fruit consumption and birth weight of new-borns.

The results of this study do not deny the effects of vegetable consumption before and during pregnancy on birth weight. One of the reasons for the absence of any association between vegetable consumption and birth weight of new-borns/risk of LBW may have been insufficient vegetable consumption. The average vegetable consumption in the highest quartile from pre- to early pregnancy (278.6 g/d) and that from early to mid-pregnancy (258.1 g/d) were less than the recommended target value for vegetable consumption in Japan (350 g/d) [37]. This may indicate that the extent of vegetable consumption in this study was insufficient to affect birth weight of new-borns/risk of LBW, even in the highest quartile. Furthermore, the difference in average vegetable consumption between the lowest and highest quartiles from pre- to early pregnancy and that from early to mid-pregnancy were 219.32 g/d and 195.6 g/d, respectively (Table S2, S4). In a mother-infant cohort study conducted in Spain, the difference in average vegetable consumption between the lowest and highest quartiles was 321.9 g/d [25], which is larger than the difference identified in our study. This Spanish study showed that women in the highest quartile of vegetable consumption during the first trimester tended to have heavier new-borns compared to women in the lowest quartile.

Regarding fruits, the differences in average consumption from pre- to early pregnancy and that from early to mid-pregnancy between the lowest and highest quartiles were 290.88 g/d and 270.53 g/d, respectively (Table S1, S3). The difference in average fruit consumption between the lowest and highest quartiles in the Spanish mother-infant cohort study was 537.4 g/d [25], and that of a birth cohort study in Denmark was 283.2 g/d [22]. The study in Spain reported that women with the highest quartile of fruit consumption during the first trimester tended to have heavier new-borns. Also, the Danish study suggested a positive association between fruit consumption and birth weight. Those results may have shown that large differences in average fruit consumption among participants were one of the reasons for positive associations.

Fruit consumption from pre- to early pregnancy and that from early to mid-pregnancy was associated with 16.9 g and 10.3 g heavier birth weight per quartile increase, respectively. These results imply that the weight birth differences between the highest and the lowest quartile were approximately 50 g and 30 g. The difference in birth weight of 20 g may be related to the fact that fruit consumption from early to mid-pregnancy were associated with new-born birth weight but not with LBW risk. This may be due to the information lost by converting continuous variable of birth weight to categorical variable of LBW risk.

In the present study, fruit consumption from early to mid-pregnancy was negatively associated with preterm birth risk (Table S7). This may suggest that a non-random sub-sample such as new-borns with LBW related to preterm birth was excluded from the analysis, as we excluded non-full-term new-borns. However, the analysis of the results suggested that including preterm birth new-borns may not strengthen but weaken the association between fruit consumption, and birth weight/LBW risk. Therefore, our results on this were not overestimated.

This study had several limitations. First, dietary consumption may have included measurement errors because each such consumption was calculated from self-reported FFQ data. Second, dietary consumption before pregnancy and early pregnancy could not be distinguished because the results of the first FFQ were calculated from dietary consumption reported for the past year from early pregnancy. However, the first FFQ largely reflected consumption before pregnancy because that questionnaire was given at early pregnancy and queried the frequency of dietary item consumption over the past year. Third, women with high fruit or vegetable consumption may maintain healthy lifestyle behaviours, and such behaviours may have been confounding. To rule out this effect, information regarding identified confounders, which were gathered from previous reports in the literature, was used to adjust the multivariable model. Fourth, the first and second FFQs were modified versions of an FFQ used in the Japan Public Health Center-Based study (JPHC study) [38–40]. We added the response option “constitutionally unable to eat it” to the FFQ used in the JPHC study. The FFQ used in that study was not validated in pregnant women; however, this was the case in the Japanese population [38–40]. Fifth, potential recall bias was may exist in answers of the two FFQs because of differing time periods for recall of the first and second FFQs. However, the first and second FFQs asked same questions about frequency and quantity, and there was no reason for under-reporting dietary consumption from early to mid-pregnancy by the difference of recall periods. Therefore, these effects are considered to be small.

Nevertheless, one of the major strengths of this study is that it investigated not only consumption during pregnancy but also that before pregnancy using two FFQs conducted at different periods. It was possible to evaluate changes in fruit and vegetable consumption before and during pregnancy and birth weight of new-borns. Moreover, this study was based on TMM BirThree Cohort Study, which was a prospective birth cohort design with a large sample size. Further, the data concerning birth weight were collected from reliable clinical birth records.

## Conclusions

The present study highlighted the association between high fruit consumption before and during pregnancy with increased birth weight of new-borns and decreased risk of LBW. Increasing the amount of fruit consumed by women with otherwise low fruit intake may contribute to increasing the birth weight of new-borns and preventing LBW. Also, the identified associations involving vegetable consumption before and during pregnancy and birth weight/risk of LBW cannot be denied by the results of this study. Further quantitative observational studies of fruit and vegetable consumption from before to during pregnancy are ultimately necessary to confirm the effect of continuous fruit and vegetable consumption on birth weight of new-borns and risk of LBW.

## Supplementary information

**Additional file 1: Table S1.** Characteristics of participants by fruit consumption from pre- to early pregnancy. **Table S2.** Characteristics of participants by vegetable consumption from pre- to early pregnancy. **Table S3.** Characteristics of participants by fruit consumption from early to mid-pregnancy. **Table S4.** Characteristics of participants by vegetable consumption from early to mid-pregnancy. **Table S5.** Energy-adjusted nutrient consumption from pre- to mid-pregnancy and new-born birth weight. **Table S6.** Fruit consumption from pre- to mid-pregnancy and new-born birth weight adjusted by nutrient consumption. **Table S7.** Energy-adjusted fruit and vegetable consumption from pre- to mid-pregnancy and preterm birth risk.

## Data Availability

The datasets analysed in this study are available from the corresponding author on reasonable request.

## Abbreviations

BMI: Body mass index
FFQ: Food frequency questionnaire
JPHC study: Japan Public Health Center-Based study
LBW: Low birth weight
95% CI: Confidence interval
OR: Odds ratio
MICE: Multivariate imputation methods by chained equations
SD: Standard deviation
TMM BirThree Cohort Study: Tohoku Medical Megabank Project Birth and Three-Generation Cohort Study

## References

[1] Katz J, Lee ACC, Kozuki N, Lawn JE, Cousens S, Blencowe H (2013). Mortality risk in preterm and small-for-gestational-age infants in low-income and middle-income countries: a pooled country analysis. Lancet..

[2] World Health Organization (2018). United Nations Children’s Fund. Survive and thrive: transforming care for every small and sick newborn.

[3] Barker DJ (2004). The developmental origins of chronic adult disease. Acta Paediatr Suppl.

[4] Nilsson PM, Ostergren PO, Nyberg P, Söderström M, Allebeck P (1997). Low birth weight is associated with elevated systolic blood pressure in adolescence: a prospective study of a birth cohort of 149378 Swedish boys. J Hypertens.

[5] Mu M, Wang SF, Sheng J, Zhao Y, Li HZ, Hu CL (2012). Birth weight and subsequent blood pressure: a meta-analysis. Arch Cardiovasc Dis.

[6] OECD (2019). Family database – OECD.

[7] Low birthweight – UNICEF DATA. Unicef, New York City. 2019. https://data.unicef.org/topic/nutrition/low-birthweight/. Accessed 16 Mar 2020.

[8] Kjøllesdal MKR, Holmboe-Ottesen G (2014). Dietary patterns and birth weight-a review. AIMS Public Health.

[9] Murphy MM, Stettler N, Smith KM, Reiss R (2014). Associations of consumption of fruits and vegetables during pregnancy with infant birth weight or small for gestational age births: a systematic review of the literature. Int J Women's Health.

[10] Cogswell ME, Parvanta I, Ickes L, Yip R, Brittenham GM (2003). Iron supplementation during pregnancy, anemia, and birth weight: a randomized controlled trial. Am J Clin Nutr.

[11] Siega-Riz AM, Hartzema AG, Turnbull C, Thorp J, McDonald T, Cogswell ME (2006). The effects of prophylactic iron given in prenatal supplements on iron status and birth outcomes: a randomized controlled trial. Am J Obstet Gynecol.

[12] Rao S, Yajnik CS, Kanade A, Fall CH, Margetts BM, Jackson AA (2001). Intake of micronutrient-rich foods in rural Indian mothers is associated with the size of their babies at birth: Pune maternal nutrition study. J Nutr.

[13] Christian P, Khatry SK, Katz J, Pradhan EK, LeClerq SC, Shrestha SR (2003). Effects of alternative maternal micronutrient supplements on low birth weight in rural Nepal: double blind randomised community trial. BMJ..

[14] Merialdi M, Carroli G, Villar J, Abalos E, Gülmezoglu AM, Kulier R (2003). Nutritional interventions during pregnancy for the prevention or treatment of impaired fetal growth: an overview of randomized controlled trials. J Nutr.

[15] Mathews F, Yudkin P, Neil A (1999). Influence of maternal nutrition on outcome of pregnancy: prospective cohort study. BMJ..

[16] Whittaker P, Tufaro PR, Rader JI (2001). Iron and folate in fortified cereals. J Am Coll Nutr.

[17] Weisman CS, Misra DP, Hillemeier MM, Downs DS, Chuang CH, Camacho FT (2011). Preconception predictors of birth outcomes: prospective findings from the Central Pennsylvania women's health study. Matern Child Health J.

[18] Jang W, Kim H, Lee BE, Chang N (2018). Maternal fruit and vegetable or vitamin C consumption during pregnancy is associated with fetal growth and infant growth up to 6 months: results from the Korean mothers and Children's environmental health (MOCEH) cohort study. Nutr J.

[19] Balázs P, Rákóczi I, Grenczer A, Foley KL (2014). Birth-weight differences of Roma and non-Roma neonates--public health implications from a population-based study in Hungary. Cent Eur J Public Health.

[20] Loy SL, Marhazlina M, Azwany YN, Hamid Jan JM (2011). Higher intake of fruits and vegetables in pregnancy is associated with birth size. Southeast Asian J Trop Med Public Health.

[21] Hassan NE, Shalaan AH, El-Masry SA (2011). Relationship between maternal characteristics and neonatal birth size in Egypt. East Mediterr Health J.

[22] Mikkelsen TB, Osler M, Orozova-Bekkevold I, Knudsen VK, Olsen SF (2006). Association between fruit and vegetable consumption and birth weight: a prospective study among 43,585 Danish women. Scand J Public Health.

[23] Kanade AN, Rao S, Kelkar RS, Gupte S (2008). Maternal nutrition and birth size among urban affluent and rural women in India. J Am Coll Nutr.

[24] Petridou E, Stoikidou M, Diamantopoulou M, Mera E, Dessypris N, Trichopoulos D (1998). Diet during pregnancy in relation to birthweight in healthy singletons. Child Care Health Dev.

[25] Ramón R, Ballester F, Iñiguez C, Rebagliato M, Murcia M, Esplugues A (2009). Vegetable but not fruit intake during pregnancy is associated with newborn anthropometric measures. J Nutr.

[26] Skreden M, Bere E, Sagedal LR, Vistad I, Øverby NC (2017). Changes in fruit and vegetable consumption habits from pre-pregnancy to early pregnancy among Norwegian women. BMC Pregnancy Childbirth.

[27] Crozier SR, Robinson SM, Godfrey KM, Cooper C, Inskip HM (2009). Women's dietary patterns change little from before to during pregnancy. J Nutr.

[28] Kuriyama S, Yaegashi N, Nagami F, Arai T, Kawaguchi Y, Osumi N (2016). The Tohoku medical megabank project: design and Mission. J Epidemiol..

[29] Kuriyama S, Metoki H, Kikuya M, Obara T, Ishikuro M, Yamanaka C (2020). Cohort Profile: Tohoku Medical Megabank Project Birth and Three-Generation Cohort Study (TMM BirThree Cohort Study): rationale, progress and perspective. Int J Epidemiol.

[30] Ministry of Education, Culture, Sports, Science and Technology: Standard tables of food composition in japan fifth revised and enlarged edition −2005- (Japanese). https://www.mext.go.jp/b_menu/shingi/gijyutu/gijyutu3/toushin/05031802.htm (2005). Accessed 6 Apr 2020.

[31] Willett W, Stampfer MJ (1986). Total energy intake: implications for epidemiologic analyses. Am J Epidemiol.

[32] ICD10data.com. 2020 ICD-10-CM Diagnosis Code P07.1. 2018. https://www.icd10data.com/ICD10CM/Codes/P00-P96/P05-P08/P07-/P07.1. Accessed 30 Mar 2020.

[33] Buuren SV. Mice: multivariate imputation by chained equations 2020. R package version 3.8.0. https://cran.r-project.org/web/packages/mice/mice.pdf. Accessed 30 Mar 2020.

[34] van Gool JD, Hirche H, Lax H, De Schaepdrijver L (2018). Folic acid and primary prevention of neural tube defects: a review. Reprod Toxicol.

[35] Grieger JA, Grzeskowiak LE, Clifton VL (2014). Preconception dietary patterns in human pregnancies are associated with preterm delivery. J Nutr.

[36] Pathirathna ML, Sekijima K, Sadakata M, Fujiwara N, Muramatsu Y, Wimalasiri KMS (2017). Impact of second trimester maternal dietary intake on gestational weight gain and neonatal birth weight. Nutrients..

[37] The Food and Agriculture Organization: Food-based dietary guidelines – Japan. http://www.fao.org/nutrition/education/food-based-dietary-guidelines/regions/countries/japan/en/. Accessed 17 Mar 2020.

[38] Kobayashi M, Sasaki S, Kawabata T, Hasegawa K, Tsugane S (2003). Validity of a self-administered food frequency questionnaire used in the 5-year follow-up survey of the JPHC study cohort I to assess fatty acid intake: comparison with dietary records and serum phospholipid level. J Epidemiol.

[39] Sasaki S, Takahashi T, Iitoi Y, Iwase Y (2003). Kobayashi M, Ishihara J, et al. food and nutrient intakes assessed with dietary records for the validation study of a self-administered food frequency questionnaire in JPHC study cohort I. J Epidemiol..

[40] Tsugane S, Kobayashi M, Sasaki S (2003). Validity of the self-administered food frequency questionnaire used in the 5-year follow-up survey of the JPHC study cohort I: comparison with dietary records for main nutrients. J Epidemiol..

